# Simultaneous Assessment of Intracranial Artery and Paravascular CSF Pulsation Using 3D Whole‐Brain Diffusion‐Prepared Cine bSSFP (DECAF) MRI

**DOI:** 10.1002/mrm.70381

**Published:** 2026-04-10

**Authors:** Chang Ni, Xiangjian Hou, SeyyedKazem HashemizadehKolowri, Seong‐Eun Kim, Qiuting Wen, Nicholas A. Frost, J. Scott McNally, Chun Yuan, Xiaodong Ma

**Affiliations:** ^1^ Department of Biomedical Engineering University of Utah Salt Lake City Utah USA; ^2^ Department of Radiology and Imaging Sciences University of Utah Salt Lake City Utah USA; ^3^ Department of Electrical and Computer Engineering University of Utah Salt Lake City Utah USA; ^4^ Department of Radiology and Imaging Sciences Indiana University School of Medicine Indianapolis Indiana USA; ^5^ Weldon School of Biomedical Engineering Department Purdue University West Lafayette Indiana USA; ^6^ Department of Neurology University of Utah Salt Lake City Utah USA

**Keywords:** artery pulsation, DECAF, diffusion‐prepared bSSFP, glymphatic system, neurofluids, paravascular CSF

## Abstract

**Purpose:**

To propose a noninvasive and quantitative MRI approach to simultaneously assessing intracranial artery pulsation and paravascular cerebrospinal fluid (CSF) pulsation in the human brain.

**Methods:**

We developed a 3D whole‐brain Diffusion‐prepared Cine bSSFP (DECAF) MRI technique, with improved motion‐sensitized driven‐equilibrium providing blood suppression and diffusion weighting. 3D golden‐angle radial trajectory, retrospective pulse gating, and GRASP image reconstruction were employed to generate cine images of 25 phases. Semi‐automatic image processing pipelines were established to quantify the arterial wall pulsatility index and the paravascular CSF pulsatility index. The ADC quantification was corrected using an analytical approach to remove T1 and T2 effects based on bSSFP. The efficacy of our proposed technique was validated on six healthy participants.

**Results:**

The developed MRI sequence was able to generate high‐resolution whole‐brain cine images with 25 cardiac phases in approximately 8 min. Reliable arterial wall and paravascular CSF pulsatility indices were quantified in the human brain with good scan–rescan reproducibility. A strong temporal relationship between those two pulsations was observed in the six participants. There is a spatial relationship between CSF pulsation and distance to arteries. In addition, the arterial wall and paravascular CSF pulsation indices are significantly correlated.

**Conclusion:**

We developed and demonstrated a DECAF MR technique, combined with an automatic image processing pipeline, enabling simultaneous quantification of intracranial artery pulsation and paravascular CSF pulsation across the cardiac cycle. This technique can be a noninvasive and quantitative imaging tool for investigating the relationship between vascular or aging‐related diseases and glymphatic dysfunction.

## 1 Introduction

The glymphatic system is a brain‐wide network that plays a critical role in facilitating the clearance of metabolic waste products from the brain parenchyma, through cerebrospinal fluid (CSF) and solute transport along the paravascular spaces [1, 2, 3]. Dysfunction of the glymphatic system has been linked to various neurodegenerative diseases. In Alzheimer's disease, impaired glymphatic function may contribute to amyloid‐β and tau accumulation [4, 5]; and in Parkinson's disease, it may affect α‐synuclein clearance [6, 7]. Recently, it was reported that glymphatic dysfunction is associated with normal pressure hydrocephalus [8].

The physiological artery pulsation is considered the primary driving force for CSF movement. Recent studies suggested augmented artery pulsation can induce abnormal paravascular CSF dynamics that impair the glymphatic system [9, 10]. In particular, one study revealed that CSF flow speed is spatially associated with distance from arteries, and hypertension would reduce arterial pulsation and compromise CSF flow [9]. Another study reported that Cerebral Amyloid Angiopathy (CAA)‐related arterial stiffness can cause paravascular clearance impairment in an AD mouse model [10]. A more recent MRI study [11] confirmed a temporal coupling of fMRI and paravascular CSF pulsation in the human brain. Despite those pilot studies, the association between artery pulsation and CSF dynamics in the human brain has not been fully established, mainly due to the lack of noninvasive and efficient approaches to characterize both arterial wall pulsation and CSF dynamics.

Dynamic MRI provides a noninvasive way to visualize vascular and CSF changes in vivo. High velocity encoding (VENC) 2D phase‐contrast or 4D flow MRI has been used to evaluate arterial pulsation by quantifying blood velocity pulsatility index [12, 13], and low‐VENC phase contrast [14] MRI or dynamic diffusion‐weighted [15] MRI have been applied to evaluate CSF dynamics. However, measuring both arterial pulsation and CSF dynamics with separate MRI sequences will take a long scan time and cause misalignment. More recently, dual‐VENC 4D flow MRI was employed to measure both blood flow and CSF flow pulsation [16]. Nonetheless, the velocity or flow pulsation of the blood is an indirect measure of arterial wall pulsation, failing to provide direct insight into the relationship between vasomotion and CSF motion. Another limitation of 4D flow is that it requires a high‐performance gradient system to achieve a small VENC value (< 5 cm/s), to accurately measure CSF flow with an intrinsically low speed.

To overcome the limitations of the existing imaging methods, we propose a novel 3D Whole‐brain Diffusion‐prepared Cine bSSFP (DECAF) MRI sequence. This sequence enables visualization of the intracranial vasomotion, as well as acquisition of low b‐value diffusion‐weighted images at different cardiac phases, which are required for assessing CSF pulsation. bSSFP acquisition is used to achieve high CSF contrast, high SNR, and low geometric distortion. To compensate for the effect of T1 and T2 relaxation on apparent diffusion coefficient (ADC) quantification from bSSFP signals [17, 18], we apply a correction of CSF ADC based on an equation derived from Bloch simulation. In addition, we develop a semi‐automatic post‐processing pipeline to quantify the pulsatility indexes of intracranial artery and paravascular CSF based on the 3D cine images. The efficacy of the proposed technique is validated on six healthy volunteers.

## 2 Methods

### 2.1 3D Diffusion‐Prepared Cine bSSFP (DECAF) MRI Sequence

**FIGURE 1:**
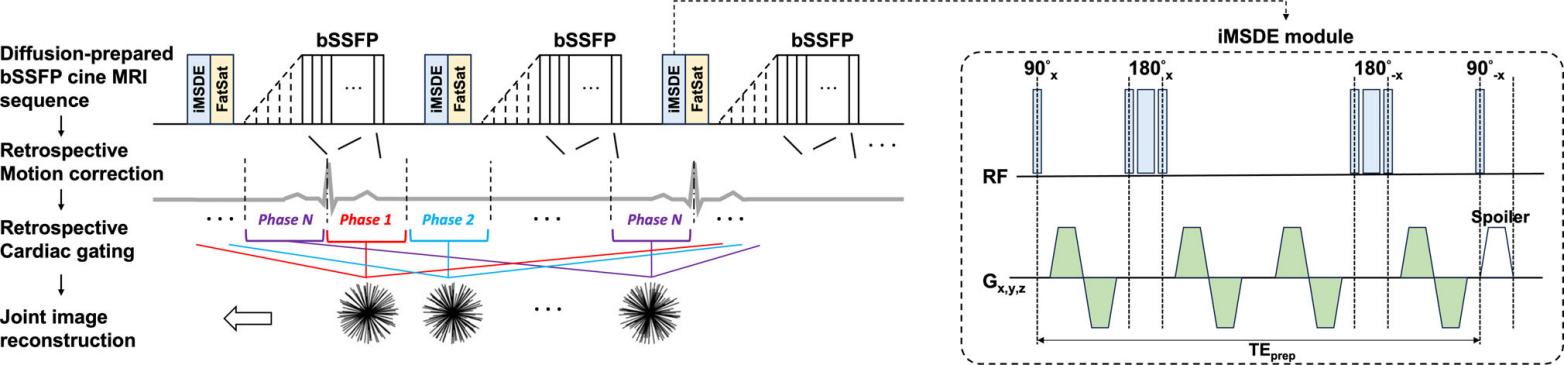
Diagram of the pulse sequence and image reconstruction for our proposed 3D Whole‐brain Diffusion‐prepared Cine bSSFP (DECAF) MRI. The sequence comprises (1) improved Motion‐Sensitized Driven‐Equilibrium (iMSDE) [19, 20] preparation for black‐blood contrast as well as diffusion weighting [21, 22], (2) fat saturation module, and (3) bSSFP acquisition using golden‐angle 3D radial sampling. Retrospective motion correction and cardiac gating will be applied to the k‐space data, and then images of different cardiac phases will be reconstructed jointly.

We developed a 3D diffusion‐prepared cine balanced steady‐state free precession (bSSFP) MRI sequence that enables simultaneous visualization of cerebral arterial wall pulsation and CSF pulsation in the whole brain. As shown in Figure 1, the sequence employs an improved Motion‐Sensitized Driven‐Equilibrium (iMSDE) [19, 20] module for blood suppression and diffusion preparation. Compared with the conventional iMSDE, bipolar gradients were used to compensate for first and second‐order motion and to reduce eddy currents [21, 22]. bSSFP acquisition is used to enhance SNR and CSF contrast. A 3D golden‐angle radial (a.k.a., 3D kooshball) trajectory is employed, which provides homogeneous k‐space coverage for flexible retrospective cardiac phase binning. This homogeneous coverage ensures uniformly distributed sampling points in k‐space, which improves image quality and enables consistent reconstruction across all cardiac phases. In addition, its inherent self‐navigator allows correction of non‐physiological head motion.

### 2.2 Image Reconstruction

The reconstruction procedure of DECAF images is also illustrated in Figure 1, with the implementation details described below:

1. *Retrospective motion correction*: A self‐navigator with approximately spatial resolution of 3 mm was reconstructed from every 1088 radial spokes (corresponding to ∼8 s). Subsequently, all low‐resolution images were registered using FSL [23] to extract the translational and rotational parameters in the image domain, which were converted to the corresponding motion parameters in the k‐space domain. Then the motion parameters were applied to the data of each k‐space spoke to generate the motion‐corrected k‐space data.
2. *Retrospective cardiac gating*: The cardiac cycles were determined through peak detection on the pulse waveform, and then the motion‐corrected k‐space data were split into 25 phases based on the time stamps.
3. *Joint image reconstruction*: The images of all phases were jointly reconstructed using GRASP [24, 25], with empirical regularization parameters of $\lambda_{\mathrm{TTV}}=0.01$ and $\lambda_{\mathrm{TV}}=0.001$. The KWIC [26] method with five connecting rings was employed for k‐space data sharing across temporal frames, improving temporal resolution while maintaining imaging quality. The equivalent acceleration factor for each phase is around 5. The *b* = 0 images were reconstructed using CG‐SENSE [27] by combining all k‐space data (9984 spokes). In contrast, substantially more spokes (49984) were acquired for the *b* = 40 data to compensate for signal attenuation introduced by iMSDE preparation.

All image reconstruction steps were conducted using MATLAB (MathWorks, Natick, MA) on a Linux computer equipped with 4 NVIDIA A100 (80GB RAM) GPUs. The image reconstruction time for each subject was ∼8 h.

### 2.3 ADC Quantification and Validation

The SSFP‐based diffusion‐prepared MRI signals exhibit additional attenuation influenced by T1 and T2 values of the tissue [28]. This effect can be ignored when using a large *b*‐value [29, 30]. However, a lower *b*‐value is required for CSF motion assessment, so the ADC quantification will be biased and must be corrected. According to the analytical simulation, in our proposed DECAF MRI sequence, the corrected ADC value can be obtained with the following equation:

(1) $${\mathrm{ADC}}_{\text{corrected}}=\frac{\ln\left(1+O_{k}O_{B}\right)-\ln\left(O_{k}\right)}{b}$$

where *O*_*k*_ and *O*_*B*_ are mathematical operators to shorten the equation, *O*_*B*_ = *f*(*T*1, *T*2, *TEp*, *trc*, *d*, *TR*, alpha), *O*_*k*_ = *f*(*ADC*_*nominal*_, *T*1, *T*2, *TEp*, *trc*, *d*, *TR*, alpha). The detailed analytical simulation is described in the Appendix A.

### 2.4 Phantom Experiments for ADC Validation

To validate the accuracy of our ADC measurements, we conducted validation experiments using a National Institute of Standards and Technology (NIST)–traceable system phantom (CaliberMRI, Boulder, CO, USA), on a 3 Tesla scanner (Prisma‐fit, Siemens Healthcare, Erlangen, Germany) with a 32‐channel head coil. The phantom was placed in the scanner room 24 h prior to scanning to ensure temperature equilibration with the environment. It was then scanned at room temperature (20°C ± 0.5°C), with the T1 layer as the imaging plane at the isocenter of the scanner (Figure S2).

We acquired the diffusion‐prepared cine bSSFP (DECAF) MRI data using our proposed sequence. The imaging parameters include TR/TE = 5.58/2.79 ms, flip angle = 45°, FOV = 200 × 200 × 200 mm^3^, acquisition matrix = 240 × 240 × 240, TE_prep_ = 52 ms, Fat Sat time *t*_fs_ = 16 ms, spoiler gradient time *t*_spoiler_ = 6 ms, number of segments for each diffusion preparation = 32, number of spokes = 49 984 for diffusion‐weighted data (*b* = 40 s/mm^2^) and 9984 for non‐diffusion‐weighted (i.e., *b* = 0) data, with a total acquisition time of 7 min 48 s. The *b* = 40 and *b* = 0 images were reconstructed separately using NUFFT [31] from the full k‐space data by combining all radial spokes. In addition, 2D quantitative mapping sequences were applied to obtain the *T*1 and *T*2 values: the T1 mapping utilized a vendor‐provided Look‐Locker sequence (TR = 10 000 ms, TE = 1.43 ms), and T2 mapping employed a multi‐echo spin echo sequence (TR = 4000 ms) with 32 echo times ranging from 50 to 1600 ms.

The corrected ADC maps were calculated based on Equation (1). For reference measurements of ADC, we acquired diffusion‐weighted data using a standard 2D single shot echo‐planar imaging (EPI) sequence (*b* = 0, 500 s/mm^2^). A larger b value, rather than the same b value, which equals 40 s/mm^2^, was selected on the 2D EPI‐DWI sequence to obtain stronger diffusion weighting and more reliable ADC measurements for reference.

Region‐of‐interest (ROI) analyses were performed on each phantom vial for both our proposed sequence and the reference EPI‐DWI, by comparing the ADC values derived from our DECAF sequence and the clinical EPI‐DWI sequence, and calculating errors before and after correction by dividing ADC values before and after correction minus EPI‐ADC by EPI‐ADC.

### 2.5 Human Data Acquisition

To verify the efficacy of our proposed MRI sequence on characterization of cerebral artery and CSF pulsation, we collected data on 6 healthy volunteers (3 male, 3 female, age range 18–71 years, mean age 41.8 years, see detailed demographic information in Table S1). The proposed sequence was used to acquire the DECAF images, with the scanner, coil, and imaging parameters the same as the phantom experiments (see Section 2.4). Note that we used a *b*‐value of 40 s/mm^2^ to maintain the signal‐to‐noise ratio of vessel wall, while achieving sufficient blood suppression [32]. Peripheral pulse waveform was recorded using a vendor‐provided oximeter. Additionally, a 3D brain time‐of‐flight (TOF) MRI dataset was acquired to obtain an anatomical reference of the cerebral arteries.

To evaluate the scan‐rescan reproducibility of our measurements, we conducted repeated scans on two participants with a 6‐day interval each using identical acquisition parameters at the same time of the day. We assessed the inter‐scan reproducibility by calculating intra‐class coefficients (ICC) of variation across the two scanning sessions.

All procedures of human experiments were approved by the Institutional Review Board in our institute. All subjects gave written informed consent before participating in the study.

### 2.6 Image Processing

#### 2.6.1 Artery Segmentation

**FIGURE 2:**
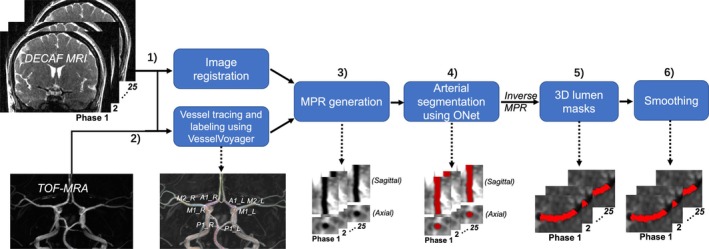
Image processing pipeline for intracranial artery segmentation. The workflow begins with automatic TOF‐DECAF registration, followed by manual centerline extraction of major cerebral arteries (M1, M2, A1, and P1). Multiplanar reconstruction (MPR) images are then generated by straightening artery along the centerline. After lumen segmentation using ONet model, 3D lumen masks are reconstructed using inverse MPR and morphological refinement.

To characterize cerebral arterial wall pulsation, the arterial lumen at each cardiac phase was segmented with the following steps (Figure 2): (1) Imaging Registration: Automatic 3D rigid registration between 3D TOF and DECAF MRI was performed using SimpleITK [33], with the same registration applied across all cardiac phases [34]. (2) Centerline extraction: Manual extraction of the centerline of major cerebral arteries around Circle of Willis (M1, M2, A1, and P1) was conducted on 3D TOF using VesselVoyager [35]. (3) Multiplanar reconstruction: 2D multiplanar reconstruction (MPR) images were generated for each intracranial artery by straightening the artery along the centerline [34]. (4) Lumen segmentation: The arterial lumen was automatically segmented on 2D MPR images using the AI model, ONet [36]. (5) 3D mask reconstruction: A 3D lumen mask was created by inverse MPR operations, followed by removal of isolated noise components using connected component analysis [37] and boundary smoothing through segmentation gap filling with a hybrid morphological filter combining 3D closing operations with adaptive region growing and slice‐wise flood‐fill processing [38]. Note that on each hemisphere of the brain, one M2 artery segment was selected to simplify subsequent analyses.

#### 2.6.2 Paravascular CSF Segmentation

**FIGURE 3:**
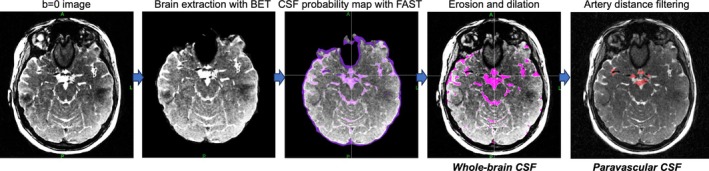
Automatic pipeline for segmenting whole‐brain and paravascular CSF. Firstly, the whole‐brain CSF masks were segmented using BET and FAST functions in FSL, followed by erosion and dilation to remove false segmentation due to banding artifacts and partial volume effects. Then, paravascular CSF regions were defined as areas surrounding the vessel boundaries within a distance threshold of 3 mm.

The Paravascular CSF was segmented with an automatic pipeline (Figure 3). Firstly, the whole‐brain CSF masks were segmented using BET and FAST functions in FSL [23], followed by erosion and dilation to remove false segmentation due to banding artifacts and partial volume effects. Then, paravascular CSF regions were defined as areas surrounding the vessel boundaries within a distance threshold. Based on established anatomical relationships, which show that paravascular spaces are typically comparable to vessel diameter [9], we used a 3 mm distance threshold determined through three‐dimensional Euclidean distance transformation. In addition, a series of distance thresholds (0.5∼6.5 mm, 0.5 mm steps) were applied to observe the layer‐specific CSF pulsation differences (see Section 2.6.4). Since the shape of paravascular space may change during the cardiac cycle, the 25 cardiac phases were processed separately and individual paravascular CSF masks were generated for each phase.

#### 2.6.3 Quantification of Artery and Paravascular CSF Pulsations

Arterial pulsation was quantified using the Arterial Wall Pulsatility Index, defined as $∆V/\bar{V}$, where ∆V is the difference between maximum and minimum lumen volumes across all cardiac phases, and $\bar{V}$ is the average lumen volume. The measurements focused on the first and second levels of the middle cerebral artery (M1 and M2) due to their relatively large diameters for reliable volumetric analysis. Additional measurements were obtained from A1 segments of the anterior cerebral artery and P1 segments of the posterior cerebral artery.

Artery volumes were calculated by counting voxels within segmented vessel masks and multiplying by the voxel size (0.42 × 0.42 × 0.42 mm^3^).

The paravascular CSF pulsation was quantified as CSF Pulsatility Index, defined as $∆\mathrm{ADC}/\bar{\mathrm{ADC}}$, where ∆ADC represents the difference between the maximum and minimum *ADC* values across all cardiac phases, and $\bar{\mathrm{ADC}}$ is the average value of all phases. For each artery, the overall pulsatility index was calculated by first computing the value of each pixel within the corresponding paravascular CSF region and then averaging all pixels. Paravascular CSF pulsation was assessed using ADC values derived from diffusion‐weighted images and reference images (*b* = 0 s/mm^2^) for each cardiac phase. ADC maps were computed using standard mono‐exponential fitting, then corrected ADC values using Equation (1).

Paired *t*‐tests with Bonferroni correction were performed across all 28 pairwise comparisons of the eight arterial segments, with significance defined as corrected *p* < 0.05.

#### 2.6.4 Investigation of the Temporal and Spatial Relationship Between Artery and Paravascular CSF Pulsations

To investigate the temporal relationship between arterial and paravascular CSF pulsations, we displayed the waveforms of normalized artery volumes and CSF ADC values across the cardiac cycle. To investigate the spatial relationship between them, we created layer‐specific CSF masks with a 0.5 mm distance interval from the arteries and compared the CSF Pulsatility Index among different layers.

## 3 Results

### 3.1 Phantom Experiments for ADC Validation

A total of 8 phantom vials containing NiCl_2_ solutions with varying concentrations were analyzed, covering a range of *T*1 and *T*2 values from 175 to 1867 ms and from 155 to 1511 ms, respectively (Figure S2). Their reference ADC values were measured from the clinical EPI‐DWI sequence.

**FIGURE 4:**
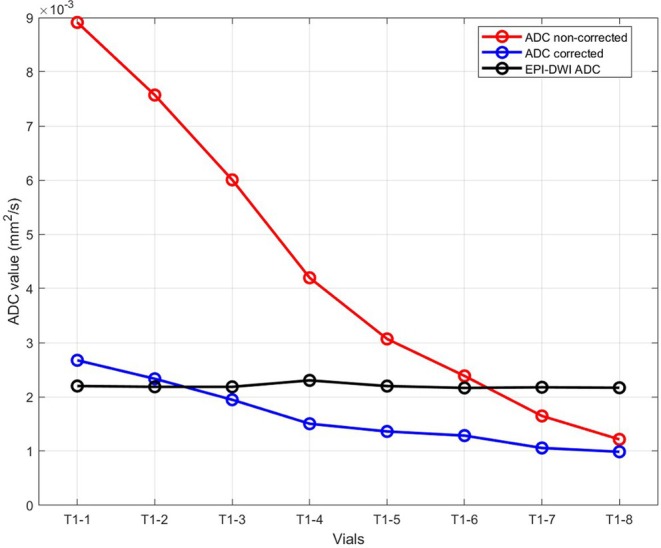
Validation of ADC quantification with phantom experiments. Shown are the ADC values calculated from our proposed diffusion‐weighted cine bSSFP (DECAF) sequence in different phantom vials (*T*1–1 to *T*1–8) before correction, after correction, as well as ADC values calculated from EPI‐DWI as a reference.

As shown in Figure 4, the corrected ADC values calculated from Equation (1) are significantly closer to the reference ADC values than non‐corrected ADC for all 8 vials with different *T*1 and *T*2 combinations (Figure 4). For the vials of large *T*1 values (*T*1–1 to *T*1–3; *T*1 > 1000 ms), the measurement errors with and without correction were 7%–22% and 176%–306%, respectively. For the vials of small *T*1 values (*T*1–4 to *T*1–8; *T*1 < 1000 ms), the errors with and without correction were 35%–55% and 10%–80%, respectively. The errors were large when *T*1 and *T*2 were significantly small because the correction accuracy is compromised in this scenario using our correction formula. Since we focus on quantifying the ADC of CSF, which has long *T*1 and *T*2 values, we will expect a small measurement error in the human data.

### 3.2 3D Whole‐Brain Cine Images for Simultaneous Artery and CSF Pulsation Visualization

**FIGURE 5:**
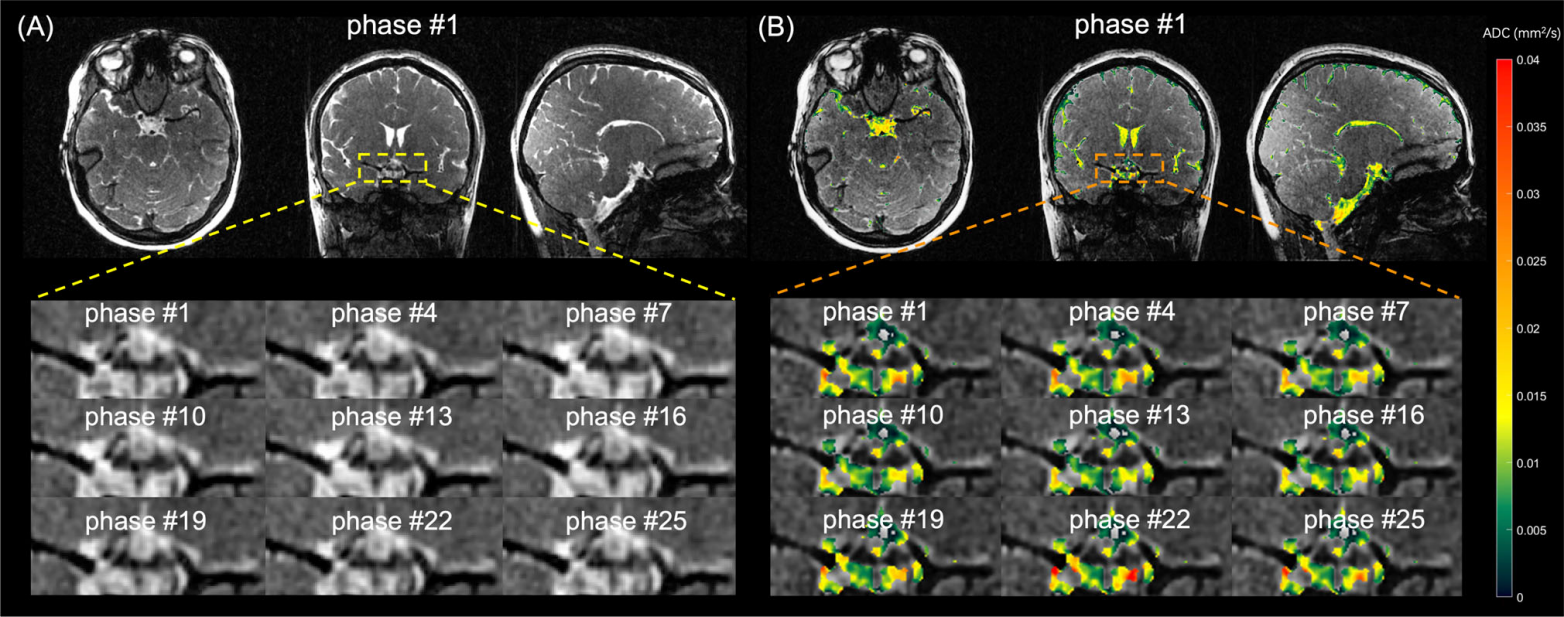
The human brain MRI results of image reconstruction and ADC quantification in one representative subject. Shown are the DECAF images (A) and quantified ADC map in the whole‐brain CSF regions overlayed on the original images (B). Images and ADC maps in every 3 phases are displayed (25 phases in total).

Using our proposed DECAF MRI sequence and image reconstruction pipeline, we generated low b‐value 3D whole‐brain diffusion‐weighted images across 25 cardiac phases, with a clear depiction of brain arterial wall‐lumen boundary and physiological pulsation (Figure 5A). The sequence can also provide ADC maps in the whole‐brain CSF regions across 25 phases, representing CSF pulsation during the cardiac cycle (Figure 5B). The *T*1 and *T*2 values of CSF were set to 4000 and 2000 ms when calculating the ADC values, as reported in the literature [39, 40]. The pulsation can be directly visualized through the movie in the Supporting Information (Figure S3).

### 3.3 Brain Artery and Paravascular CSF Segmentation

**FIGURE 6:**
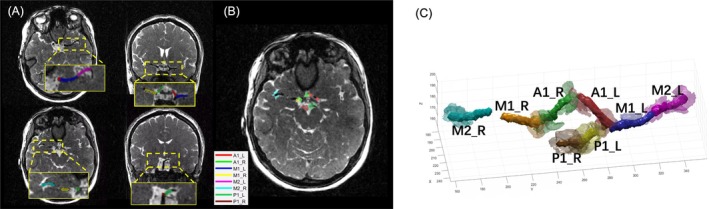
Segmentation results of major intracranial arteries and paravascular CSF in one representative subject. (A) artery segmentation for axial and coronal view, (B) paravascular CSF segmentation, and (C) 3D rendering of lumen and paravascular CSF masks for each artery.

The automated segmentation pipeline successfully identified arterial structures and paravascular CSF spaces in all participants (see the result of a representative participant in Figure 6). For all major arteries that we measured, the AI‐based segmentation achieved robust arterial inner‐wall delineation of each cardiac phase, enabling the measurement of artery volume changes throughout the cardiac cycle. The automatic paravascular CSF segmentation method effectively defined paravascular CSF spaces from bulk CSF, with clear spatial correspondence between arterial segments and their associated paravascular regions.

### 3.4 Temporal Relationship Between Arterial and Paravascular CSF Pulsation

**FIGURE 7:**
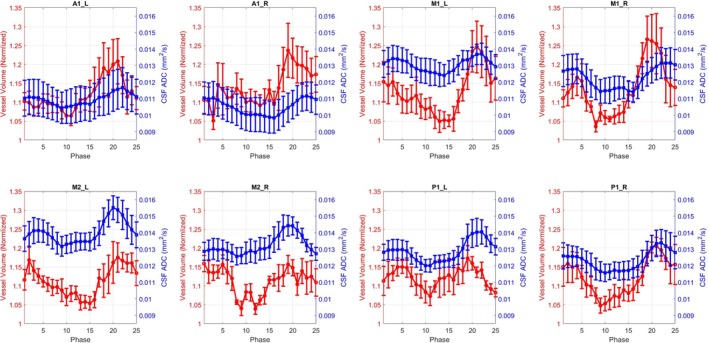
Temporal waveforms of normalized artery volumes (left axis, red curve) and the corresponding paravascular CSF ADC values (right axis, blue curve) across 25 cardiac phases, in each artery (M1, M2, A1, P1). Shown are the average values ± Standard Error of the Mean (SEM) of all 6 participants. A strong temporal coupling between artery and CSF pulsations can be observed. In addition, temporal lags can be observed between artery pulsation and paravascular CSF pulsation, with the former preceding the latter up to 3 cardiac phases. The artery volume of each vessel segment was normalized by dividing by its minimum value across the 25 cardiac phases.

The temporal waveforms revealed that the arterial wall motion and paravascular CSF dynamics are synchronized in the cardiac cycle (Figure 7), for all 6 participants. Specifically, both arterial volume and paravascular CSF ADC measurements showed systolic peak during the cardiac phases of 20 to 22, and diastolic trough during phases of 10–15. The 1D cross‐correlation coefficients between these two waveforms are larger than 0.99 for all arteries, suggesting a high temporal coupling between artery and paravascular CSF pulsation. In addition, temporal lags can be visually observed between artery pulsation and paravascular CSF pulsation, with the former preceding the latter up to 3 cardiac phases.

### 3.5 Quantitative Analysis of Arterial Wall and Paravascular CSF Pulsatility Indexes

**FIGURE 8:**
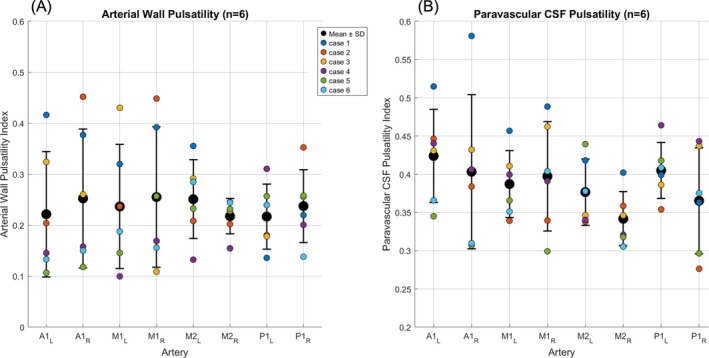
Quantitative results of (A) Arterial Wall Pulsatility Index and (B) Paravascular CSF Pulsatility Index. Shown are the individual and average values ± Standard Error of the Mean (SEM) of all 6 participants and each artery. Paired *t*‐tests with Bonferroni correction for multiple comparisons (28 pairwise comparisons) revealed no statistically significant differences across the eight arterial segments for either metric (all corrected *p* > 0.05).

The arterial wall and paravascular CSF pulsatility indexes averaged across all 6 participants for each artery are shown in Figure 8. The paravascular CSF pulsatility indexes tend to be larger in A1 and P1 compared with those in M1 and M2, possibly because the paravascular CSF surrounding A1 and P1 connects to the subarachnoid region next to large arteries such as the internal carotid artery and basilar artery, leading to an overall cardiac‐driven higher pulsatility. Paired *t*‐tests showed no statistically significant differences in the arterial wall pulsatility index (all uncorrected *p* > 0.05). For the paravascular CSF pulsatility index, no pairwise comparison reached statistical significance after Bonferroni correction (minimum corrected *p* = 0.057). Both arterial wall and paravascular CSF pulsatility indexes can be directly visualized in the 3D map (Figure S4A).

### 3.6 Reproducibility Assessment

**FIGURE 9:**
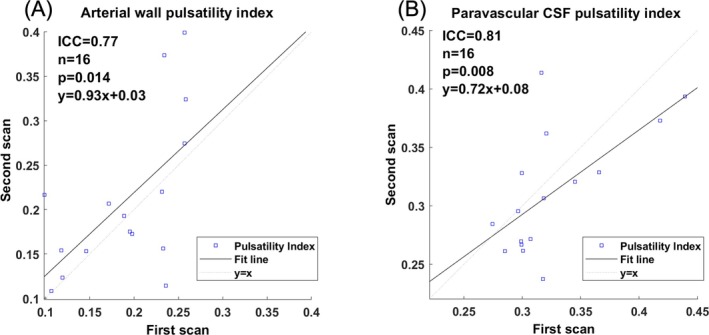
Result of scan‐rescan reproducibility test. Shown are the scatter plots of arterial wall pulsatility index (A) and paravascular CSF pulsatility index (B) for the first scan (*x*‐axis) versus the second scan (*y*‐axis) on two healthy subjects (Male, 36 and 61 years old). Both pulsatility indexes have good reproducibility (intra‐class correlation coefficient > 0.75, *p* < 0.05).

The scan‐rescan reproducibility test suggested good consistency of both arterial wall and paravascular CSF pulsatility indexes measured from the proposed DECAF MRI technique (Figure 9). The intra‐class correlation coefficients of arterial wall pulsatility index and paravascular CSF pulsatility index between two scans are 0.77 and 0.81, respectively, indicating good scan‐rescan reproducibility.

### 3.7 Spatial Relationship Between CSF Pulsation and Distance From Arteries

**FIGURE 10:**
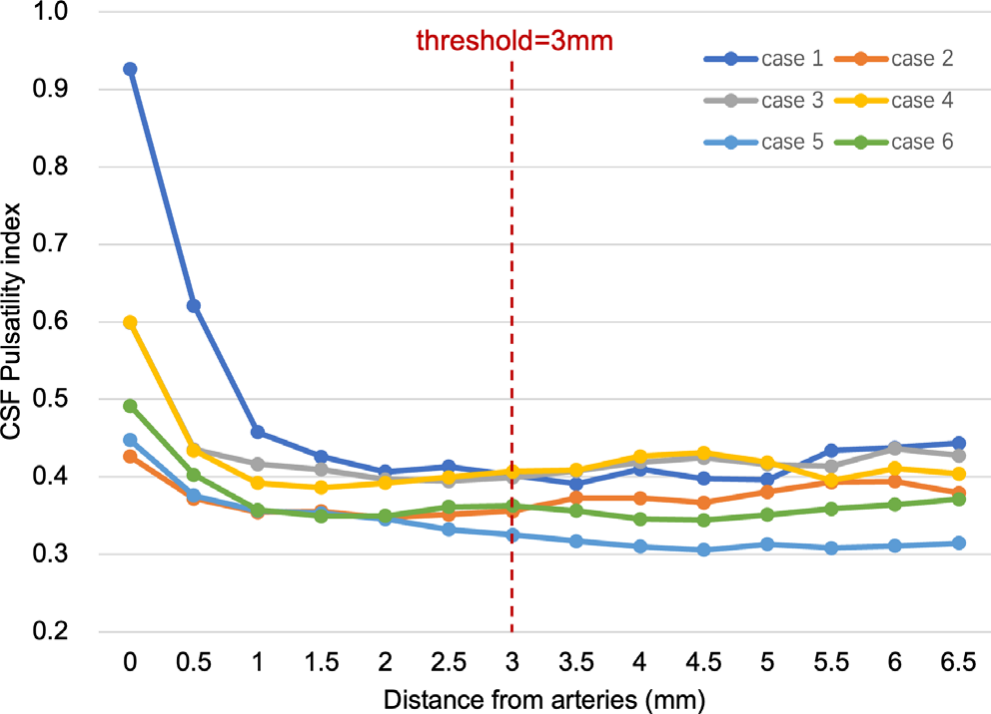
Spatial relationship between CSF pulsation and distance from arteries. Shown are the pulsatility indexes of CSF in different distances to arteries (0–6.5 mm) for each participant, averaged over all 8 arteries. The vertical dashed line at 3 mm indicates the boundary of the paravascular region defined in Figure 3. The CSF next to the arteries has the highest pulsatility index, which will decrease when the distance to arteries increases from 0 to 1.5 mm. From 1.5 to 3 mm, some participants show slightly increased pulsatility indices, while others show stable or decreased values, reflecting inter‐subject variability. Beyond 3 mm, pulsatility indices remain stable.

As shown in Figure 10, the CSF immediately adjacent to the arteries has the highest pulsatility index, which decreases as the distance to the arteries increases from 0 to 1.5 mm. From 1.5 to 3 mm, some participants show slightly increased pulsatility indices, possibly due to pulsation from nearby brain tissue or arterial segments, while others show stable or decreased values. Beyond 3 mm, the CSF pulsatility indices remain stable. Despite inter‐subject variability, the overall trend of highest CSF pulsatility near the artery wall is consistent across all six participants and can be directly visualized in the 3D CSF pulsatility index map (see Figure S4B).

## 4 Discussion

While the cardiac‐related intracranial artery pulsation is generally believed to be the driving force of paravascular CSF movement for the glymphatic transport, the evidence is limited to preclinical studies due to a lack of reliable techniques that can noninvasively characterize both artery and CSF pulsations. In this study, we have proposed and developed a diffusion‐prepared cine bSSFP (DECAF) MRI technique, which can simultaneously evaluate pulsations of intracranial arteries and paravascular CSF. Development of this technique includes novel pulse sequence design, joint image reconstruction and a semi‐automatic image processing pipeline. Results from 6 healthy subjects demonstrated that this technique can reliably quantify both arterial wall and paravascular CSF pulsatility indexes in major Circle‐of‐Willis arteries, and characterize the temporal and spatial relationship between the two pulsations.

Traditionally, the pulsation of intracranial arteries is assessed by measuring the blood velocity pulsatility index using phase contrast [41] or 4D flow MRI [42] methods. In this study, we assess arterial pulsation using arterial wall pulsatility index measured from our proposed DECAF MRI sequence, which can directly characterize the physiological vasomotion associated with paravascular CSF movement. While the blood velocity pulsatility index increases with age, we found the arterial wall pulsatility index decreases with age (Figure S5A). This is consistent with the fact that the artery stiffness can increase with age and may potentially lead to reduced vasomotion [43, 44]. Also, the arterial wall pulsatility index decreases when systolic blood pressure or pulse pressure (difference between systolic and diastolic blood pressure) increases (Figure S5BC), supporting that the arterial wall pulsatility index is closely related to artery stiffness. Note that those observations are based on a limited sample size (6 participants), and they will be further investigated in future studies.

To reliably measure arterial wall pulsatility index, accurate lumen volumes need to be measured for each cardiac phase. In this study, we first reformat each intracranial artery to 2D by straightening it along the centerline [34], and then use a deep‐learning segmentation model, ONet [45], to obtain the contour of lumen. The lumen masks obtained from ONet are transformed back to 3D and then further smoothed. Though the spatial resolution (0.8 mm) is higher than the arterial wall motion (as small as 0.2 mm for a 2 mm artery, assuming the physiological lumen change is as low as 10%), previous studies reported that this sub‐voxel movement can be accurately detected using deep learning [46]. Our simulation experiment also confirmed that ONet can differentiate lumen changes as low as 0.2 mm (Figure S7), and human results show a good scan‐rescan reproducibility of arterial wall pulsatility index measurement (Figure 9).

The concept of evaluating paravascular CSF is similar to the single‐shot EPI‐based dynamic DWI technique [15], though we employed a diffusion‐prepared cine bSSFP MRI sequence as an alternative acquisition approach. Consistent with the previous study [15], we see an age effect of paravascular CSF pulsation (Figure S6A), such as trough ADC (Figure S6B). Compared with single‐shot EPI DWI, the bSSFP MRI sequence is distortion‐free and can achieve higher spatial resolution (0.8 mm isotropic in this study), which is required for a clear delineation of artery structures. Therefore, it is more suitable for characterizing both artery pulsation and CSF pulsation.

As expected, we confirmed there is a temporal coupling between pulsation of intracranial arteries and pulsation of their surrounding CSF. The waveforms of artery volumes and paravascular CSF ADC values across the cardiac cycle follow the same temporal pattern, and they have visually observable lags (artery pulsation precedes paravascular CSF pulsation by up to 3 phases). This is consistent with recent phase contrast or 4D flow MRI‐based studies [16, 47]. However, since we measure random motion to assess CSF pulsation using low b‐value diffusion weighting, similar to the dynamic DWI [15, 48] technique, the interpretation of the lags between artery and CSF pulsations is different. Specifically, in the flow‐based measurement, the CSF exhibits a peak flow in the same direction right after the diastolic peak of blood velocity, and a peak flow in the opposite direction right after the systolic peak of blood velocity [16]. In contrast, our diffusion‐based measurement shows that CSF exhibits a peak ADC immediately following the systolic peak of artery volume, consistent with a recent human study [11].

We also observed a spatial relationship between pulsation of CSF and its distance to arteries. The CSF has the highest pulsatility index (defined as ADC pulsatility) near the arteries and will have a reduced pulsatility index when the distance to arteries increases. This distance‐dependent relationship supports the hypothesis that artery pulsation may be a primary driver of paravascular CSF movement. Interestingly, some participants showed slightly increased CSF pulsatility index when the distance to arteries ranged from 1.5 to 3 mm, possibly due to the pulsatility of brain parenchyma in those regions [49]. This indicates that parenchymal tissue pulsation may also contribute to the paravascular CSF pulsation, secondary to the vascular‐driven mechanisms.

There is a positive correlation between arterial wall pulsatility index and paravascular CSF pulsatility index (*r* = 0.29, *p* = 0.04, *N* = 48 with 8 arteries in 6 participants, Fig S6). The association is weaker than expected, suggesting a complicated relationship between vasomotion and the pulsatile motion of the paravascular CSF. Instead of the total artery volume changes, CSF pulsation may be closer related to the local artery area changes, and the relationship may also be related to heart rate which will affect the wall movement velocity [2]. Investigation of those topics requires higher image resolution and sophisticated image analysis. We also found that paravascular CSF in small arteries (M2) has a lower pulsatility index than large arteries (M1), which is consistent with the previous study [15].

One major disadvantage of DECAF sequence is the inaccurate ADC quantification. In this study, we proposed an analytical approach to remove T1 and T2 effects to obtain corrected ADC values of the CSF, assuming the T1 and T2 do not significantly change in different locations, cardiac phases, and subjects. To completely resolve this issue, T1 and T2 maps need to be acquired on each subject. Several rapid brain relaxation mapping methods have been proposed recently [50, 51] and they can be applied here. While our phantom experiments validated the accuracy of ADC quantification, in vivo ADC measurements in the human brain are influenced by additional signal attenuation induced by involuntary bulk motion, for example, the signal loss caused by motion‐induced inter‐shot phase errors, a common issue in diffusion‐prepared MRI sequences [52, 53]. This is consistent with a recent work introducing the concept of “CSF mobility” to describe signal attenuation caused by slow flow, laminar flow, or back‐and‐forth motion of CSF [54]. This bulk‐motion‐induced bias could be corrected using a stabilizer gradient during diffusion preparation or using sophisticated image reconstruction [55], which will be implemented and tested in our future work. Given this limitation, an important consideration in interpreting the in vivo ADC measurements from the proposed technique is that they reflect motion‐weighted parameters rather than pure diffusion coefficients.

Another limitation of DECAF MRI is the banding artifacts caused by bSSFP acquisition, which can induce signal dropout, especially in the A1 arteries. In the six participants we included, we did not see a significant influence of the banding artifacts on quantifying the arterial and paravascular CSF pulsation. The banding artifacts can also be reduced using phase‐cycling strategies [56].

The current implementation of image reconstruction requires approximately 8 h per subject on a high‐performance workstation. This reconstruction pipeline was developed as a research tool for proof‐of‐concept validation and has not been optimized for computational efficiency. For clinical deployment, substantial speed improvements can be achieved through integrating coil compression [57], and re‐programming the reconstruction pipeline in C++ with optimized memory management and parallel processing.

## 5 Conclusion

In conclusion, we developed a 3D Whole‐brain Diffusion‐prepared Cine bSSFP (DECAF) MR sequence, combined with an automatic image processing pipeline. This new technique enables simultaneous quantification of intracranial arterial pulsation and paravascular CSF pulsation throughout the cardiac cycle. Human results demonstrated a strong temporal and spatial correlation between arterial and paravascular CSF pulsation. The CSF immediately adjacent to the arteries has the highest pulsatility index, which decreases with increasing distance from the arteries, supporting the hypothesis that paravascular CSF dynamics are driven by cardiac‐related arterial pulsation. This noninvasive and quantitative imaging approach can be applied to investigate the relationship between vascular or aging‐related diseases and glymphatic system dysfunction.

## Funding

This work was supported by National Institutes of Health (R01‐HL103609, R01‐NS127317), Department of Radiology and Imaging Sciences and Center on Aging at University of Utah (2023 Radiology Seed Grant and Center on Aging Grant), ADRC at Utah State University (2024 ADRC Research Catalyst).

## Supporting information


**Figure S1:** Evolution of magnetization during the first 2 diffusion preparation periods in the proposed DECAF sequence.
**Figure S2:** The distribution of the vials in the phantom, along with their measured T1, T2, EPI‐DWI ADC values, and NiCl_2_ concentrations, T1, T2 values provided in the user manual.
**Table S1:** Demographic information of the participants.
**Table S2:** Arterial wall pulsatility index (PI) and paravascular CSF PI for each artery segment across all subjects.
**Table S3:** ADC maximum and minimum values (×10^−3^ mm^2^/s) across the cardiac cycle for each artery segment and each subject.
**Figure S3:** The human brain MRI results of image reconstruction and ADC quantification in one representative subject (movie version of Figure 5). Shown are the diffusion‐prepared cine bSSFP (DECAF) images (A) and quantified ADC map in the whole‐brain CSF regions overlayed on the original images (B).
**Figure S4:** 3D visualization of the paravascular CSF pulsatility index map from one representative participant. (A) Simultaneous visualization of arterial wall and paravascular CSF pulsatility indexes in the 3D space. (B) The layer‐specific paravascular CSF pulsatility index in the 3D space.
**Figure S5:** Relationship of artery wall pulsatility index with age and blood pressure.
**Figure S6:** Relationship of paravascular CSF pulsation with age and Artery Wall Pulsatility Index.
**Figure S7:** A simulation experiment to confirm the AI model can detect cerebral arterial lumen changes. A high‐resolution 2D MPR image (0.25 mm isotropic) of a 2 mm‐diameter artery was used to synthesize cine MRI images. The synthesized images have the same spatial and temporal resolution in this study (0.83 mm isotropic, 16 phases). To simulate the cardiac‐driven lumen changes, the static image was zoomed by a predefined factor to generate an image corresponding to each phase. The process was repeated with multiple ground‐truth pulsatility indices ranging from 0.05 to 0.3. The measured pulsatility indices were calculated by segmenting synthesized cine images using the ONet model. Though the measured volume changes were overestimated (likely because the measured changes were rounded by voxel size), they can well characterize the ground‐truth waveforms. The measured and ground‐truth pulsatility indices have a good consistency (ICC = 0.90).

## Data Availability

The data that support the findings of this study are available from the corresponding author upon reasonable request.

## Appendix A Correction of Apparent Diffusion Coefficient Based on Bloch Simulation

### Theory

Starting from $M_{z}^{0}=M_{0}$, the thermal‐equilibrium value for vector *M* in the presence of only *B*_0_ field in the rotating frame [58], we flip the bulk magnetization 90° along the *x*′‐axis. The subsequent bipolar diffusion gradients cause dephasing of magnetization in blood flow areas (black‐blood effect), which is beneficial for imaging vessel walls. Simultaneously, the remaining brain tissue is affected by these bipolar gradients, achieving a diffusion‐weighting effect.

After diffusion preparation, a spoiler gradient diminishes the remaining transverse magnetization. Following the fat‐saturation module, the readout sequence is executed. We set five pre‐scan pulses to achieve a balanced steady state.

Before the first pre‐scan pulse, we define this longitudinal magnetization as $M_{z}\left(1,-\right)$:

(A1)
$$M_{z}\left(1,-\right)=M_{z}^{0}\left(1-e^{-\frac{d}{T1}}\right)+M_{z}^{0}e^{-\frac{\mathrm{TEp}}{T2}}e^{-\mathrm{bD}}e^{-\frac{d}{T1}}$$

where TEp is the time for the MSDE process, d is the time interval from the last 90°‐x pulse of MSDE to the first pulse of the pre‐scan bSSFP, D is the diffusion coefficient, and b represents the *b* value calculated by the bipolar gradient's duration and strength:

(A2)
$$b=\gamma^{2}3G_{\max}^{2}\left(\frac{8\;{\mathrm{plt}}^{3}}{3}+12\;{\mathrm{plt}}^{2}\;s+\frac{46\;\mathrm{plt}\;s^{2}}{3}+\frac{92\;s^{3}}{15}\right)$$

where γ is the gyromagnetic ratio, $G_{\max}$ is the diffusion gradient strength, s equals $G_{\max}/\mathrm{SR}$, where SR is the slew rate, and plt is the plateau length of the diffusion gradient.

We denote the balanced steady‐state magnetization as $M_{\mathrm{SS}}\;\left(1,0+\right)$:

(A3)
$$M_{x^{\prime }}^{\mathrm{ss}}\left(1,0+\right)=0$$

(A4)
$$M_{y^{\prime }}^{\mathrm{ss}}\left(1,0+\right)=\frac{\;M_{z}\left(1,-\right)\left(1-E_{1}\right)\sin\alpha }{\left(1-E_{1}\cos\alpha \right)-\left(E_{1}-\cos\alpha \right)\;E_{2}}$$

(A5)
$$M_{z^{\prime }}^{\mathrm{ss}}\left(1,0+\right)=\frac{\;M_{z}\left(1,-\right)\left(1-E_{1}\right)\left(E_{2}+\cos\;\alpha \right)}{\left(1-E_{1}\cos\alpha \right)-\left(E_{1}-\cos\alpha \right)\;E_{2}}$$

After the readout, there is a time interval trc before the next diffusion preparation begins. During this period, both transverse and longitudinal magnetizations free precession toward thermal equilibrium at rates determined by T2 decay and T1 recovery. However, this time is insufficient for complete recovery to $M_{0}$. As a result, at the beginning of the next MSDE module, the magnetization components are:

(A6)
$$M_{z}^{1}=M_{z}^{0}\left(1-e^{-\frac{\mathrm{trc}}{T1}}\right)+M_{z^{\prime }}^{\mathrm{ss}}\left(0+\right)e^{-\frac{\mathrm{trc}}{T1}}$$

(A7)
$$M_{y}^{1}=M_{y^{\prime }}^{\mathrm{ss}}\left(0+\right)e^{-\frac{\mathrm{trc}}{T2}}$$

After the second MSDE and fat saturation module, we obtain $M_{z}\left(2,-\right)$, the magnetization before the second pre‐scan pulse. Note that $M_{y}$ is dephased during the MSDE process (Figure S1).

Starting from $M_{z}\left(2,-\right)$, the system undergoes bSSFP and reaches a different steady state magnetization $M_{\mathrm{SS}}\left(2,+\right)$:

(A8)
$$M_{z}\left(2,-\right)=M_{z}^{0}\left(1-e^{-\frac{d}{T1}}\right)+M_{z}^{1}e^{-\frac{\mathrm{TEp}}{T2}}e^{-\mathrm{bD}}e^{-\frac{d}{T1}}$$

(A9)
$$M_{y^{\prime }}^{\mathrm{ss}}\left(2,0+\right)=\frac{\;M_{z}\left(2,-\right)\left(1-E_{1}\right)\sin\alpha }{\left(1-E_{1}\cos\alpha \right)-\left(E_{1}-\cos\alpha \right)\;E_{2}}$$

(A10)
$$M_{z^{\prime }}^{\mathrm{ss}}\left(2,0+\right)=\frac{\;M_{z}\left(2,-\right)\left(1-E_{1}\right)\left(E_{2}+\cos\;\alpha \right)}{\left(1-E_{1}\cos\alpha \right)-\left(E_{1}-\cos\alpha \right)\;E_{2}}$$

Thus, for the nth round of diffusion preparation followed by bSSFP, the nth steady‐state magnetization $M_{\mathrm{SS}}\;\left(n,0+\right)$ is:

(A11)
$$M_{x^{\prime }}^{\mathrm{ss}}\left(n,0+\right)=0$$

(A12)
$$M_{y^{\prime }}^{\mathrm{ss}}\left(n,0+\right)=\frac{\;M_{z}\left(n,-\right)\left(1-E_{1}\right)\sin\alpha }{\left(1-E_{1}\cos\alpha \right)-\left(E_{1}-\cos\alpha \right)\;E_{2}}$$

(A13)
$$M_{z^{\prime }}^{\mathrm{ss}}\left(n,0+\right)=\frac{\;M_{z}\left(n,-\right)\left(1-E_{1}\right)\left(E_{2}+\cos\;\alpha \right)}{\left(1-E_{1}\cos\alpha \right)-\left(E_{1}-\cos\alpha \right)\;E_{2}}$$

with

(A14)
$$M_{z}\left(n,-\right)=M_{z}^{0}\left(1-e^{-\frac{d}{T1}}\right)+M_{z}^{n-1}e^{-\frac{\mathrm{TEp}}{T2}}e^{-\mathrm{bD}}e^{-\frac{d}{T1}}$$

where

(A15)
$$M_{z}^{n-1}=M_{z}^{0}\left(1-e^{-\frac{\mathrm{trc}}{T1}}\right)+M_{z^{\prime }}^{\mathrm{ss}}\left(n-1,0+\right)e^{-\frac{\mathrm{trc}}{T1}}$$

Similarly,

(A16)
$$M_{z}^{n}=M_{z}^{0}\left(1-e^{-\frac{\mathrm{trc}}{T1}}\right)+M_{z^{\prime }}^{\mathrm{ss}}\left(n,0+\right)e^{-\frac{\mathrm{trc}}{T1}}$$

Therefore, we have:

(A17)
$$M_{z}^{n}=O_{A}+M_{z}^{n-1}e^{-\mathrm{bD}}O_{B}$$

where

(A18)
$$\left\{\begin{array}{l} O_{A}=M_{z}^{0}\left(1-e^{-\frac{\mathrm{trc}}{T1}}\right)+M_{z}^{0}\left(1-e^{-\frac{d}{T1}}\right)\frac{\left(1-E_{1}\right)\left(E_{2}+\cos\;\alpha \right)}{\left(1-E_{1}\cos\alpha \right)-\left(E_{1}-\cos\alpha \right)\;E_{2}}e^{-\frac{\mathrm{trc}}{T1}}\\ O_{B}=e^{-\frac{\mathrm{TEp}}{T2}}e^{-\frac{d}{T1}}\frac{\left(1-E_{1}\right)\left(E_{2}+\cos\;\alpha \right)}{\left(1-E_{1}\cos\alpha \right)-\left(E_{1}-\cos\alpha \right)\;E_{2}}e^{-\frac{\mathrm{trc}}{T1}} \end{array}\right.$$

From the sum of geometric sequences, we get:

(A19)
$$M_{z}^{n-1}=O_{A}\frac{1-{\left(e^{-\mathrm{bD}}O_{B}\right)}^{n-1}}{1-e^{-\mathrm{bD}}O_{B}}+M_{z}^{0}{\left(e^{-\mathrm{bD}}O_{B}\right)}^{n-1}$$

From Equations (A12) and (A14), we can calculate:

$$\begin{array}{cc} M_{y^{\prime }}^{\mathrm{ss}}\left(n,0+\right) & =\left(M_{z}^{0}\left(1-e^{-\frac{d}{T1}}\right)\right.\\ & \;\left.+\left(O_{A}\frac{1-{\left(e^{-\mathrm{bD}}O_{B}\right)}^{n-1}}{1-e^{-\mathrm{bD}}O_{B}}+M_{z}^{0}{\left(e^{-\mathrm{bD}}O_{B}\right)}^{n-1}\right)e^{-\mathrm{bD}}e^{-\frac{\mathrm{TEp}}{T2}}e^{-\frac{d}{T1}}\right) \end{array}$$

(A20)
$$*\frac{\left(1-E_{1}\right)\sin\alpha }{\left(1-E_{1}\cos\alpha \right)-\left(E_{1}-\cos\alpha \right)\;E_{2}}$$

and the transverse readout magnetization amplitude after time TE is:

(A21)
$$M_{t}\left(n\right)=M_{y^{\prime }}^{\mathrm{ss}}\left(n,0+\right)e^{-\frac{\mathrm{TE}}{T2}}$$

### K‐Space Centerline Amplitude Simulation

From the relationship in Equation A17, when the steady state is reached:

(A22)
$$M_{z}^{n}=M_{z}^{n-1}=M_{z}^{\mathrm{ss}}$$

Thus, for the steady state magnitude:

(A23)
$$M_{z}^{\mathrm{ss}}=O_{A}+M_{z}^{\mathrm{ss}}e^{-\mathrm{bD}}O_{B}$$

Therefore, we obtain:

(A24)
$$M_{z}^{\mathrm{ss}}=\frac{O_{A}}{1-e^{-\mathrm{bD}}O_{B}}$$

We define the convergence error as:

$$\Delta M_{z}=M_{z}^{n}-M_{z}^{\mathrm{ss}}=O_{A}\frac{1-{\left(e^{-\mathrm{bD}}O_{B}\right)}^{n}}{1-e^{-\mathrm{bD}}O_{B}}+M_{z}^{0}{\left(e^{-\mathrm{bD}}O_{B}\right)}^{n}-\frac{O_{A}}{1-e^{-\mathrm{bD}}O_{B}}$$

(A25)
$$={\left(e^{-\mathrm{bD}}O_{B}\right)}^{n}\left(M_{z}^{0}-\frac{O_{A}}{1-e^{-\mathrm{bD}}O_{B}}\right)$$

If we normalize the error to determine the number of repetitions n required:

(A26)
$$\frac{\Delta M_{z}}{M_{z}^{\mathrm{ss}}}=\frac{{\left(e^{-\mathrm{bD}}O_{B}\right)}^{n}\left(M_{z}^{0}\left(1-e^{-\mathrm{bD}}O_{B}\right)-O_{A}\right)}{O_{A}}={\left(e^{-\mathrm{bD}}O_{B}\right)}^{n}\left(\frac{M_{z}^{0}}{M_{z}^{\mathrm{ss}}}-1\right)$$

This equation allows us to perform a simulation to determine at which n value the magnitude reaches steady state.

### ADC Correction

To simplify calculations, we define:

(A27)
$$\left\{\begin{array}{l} O_{y}=\frac{\left(1-E_{1}\right)\sin\alpha }{\left(1-E_{1}\cos\alpha \right)-\left(E_{1}-\cos\alpha \right)\;E_{2}}\\ O_{z}=\frac{\left(1-E_{1}\right)\left(E_{2}+\cos\;\alpha \right)}{\left(1-E_{1}\cos\alpha \right)-\left(E_{1}-\cos\alpha \right)\;E_{2}} \end{array}\right.$$

Thus, Equations (A12) and (A13) can now be represented as:

(A28)
$$M_{y^{\prime }}^{\mathrm{ss}}\left(n,0+\right)=M_{z}\left(n,-\right)O_{y}$$

(A29)
$$M_{z^{\prime }}^{\mathrm{ss}}\left(n,0+\right)=M_{z}\left(n,-\right)O_{z}$$

Therefore:

(A30)
$$M_{t}\left(n\right)=M_{y^{\prime }}^{\mathrm{ss}}\left(n,0+\right)e^{-\frac{\mathrm{TE}}{T2}}=M_{z}\left(n,-\right)O_{y}e^{-\frac{\mathrm{TE}}{T2}}=\left(O_{d}+M_{z}^{\mathrm{ss}}e^{-\mathrm{bD}}O_{p}\right)O_{y}e^{-\frac{\mathrm{TE}}{T2}}$$

where we introduce two more operators to shorten the equations:

(A31)
$$\left\{\begin{array}{l} O_{d}=M_{z}^{0}\left(1-e^{-\frac{d}{T1}}\right)\\ O_{p}=e^{-\frac{\mathrm{TEp}}{T2}}e^{-\frac{d}{T1}} \end{array}\right.$$

Substituting Equation A24, the readout magnitude becomes:

(A32)
$$M_{t}\left(n\right)=\left(O_{d}+\frac{O_{A}}{1-e^{-\mathrm{bD}}O_{B}}e^{-\mathrm{bD}}O_{p}\right)O_{y}e^{-\frac{\mathrm{TE}}{T2}}$$

Assuming the transverse magnetization is proportional to the acquired signal, the signals with b=0 and b≠0 are:

(A33)
$$S_{0}=\left(O_{d}+\frac{1}{1-O_{B}}O_{A}O_{p}\right)O_{y}e^{-\frac{\mathrm{TE}}{T2}}$$

(A34)
$$S_{1}=\left(O_{d}+\frac{e^{-\mathrm{bD}}}{1-e^{-\mathrm{bD}}O_{B}}O_{A}O_{p}\right)O_{y}e^{-\frac{\mathrm{TE}}{T2}}$$

where b is the non‐zero b value we use.

The nominal calculation of ADC, which is inaccurate for this sequence, is calculated as:

$${\mathrm{ADC}}_{\text{nominal}}=\frac{\ln\left(S_{0}\right)-\ln\left(S_{1}\right)}{b}$$

(A35)
$$\begin{array}{cc} & =\frac{\ln\left(\left(O_{d}+\frac{1}{1-O_{B}}O_{A}O_{p}\right)O_{y}e^{-\frac{\mathrm{TE}}{T2}}\right)-\ln\left(\left(O_{d}+\frac{e^{-\mathrm{bD}}}{1-e^{-\mathrm{bD}}O_{B}}O_{A}O_{p}\right)O_{y}e^{-\frac{\mathrm{TE}}{T2}}\right)}{b}\\ & \;=\frac{\ln\left(\frac{O_{d}+\frac{1}{1-O_{B}}O_{A}O_{p}}{O_{d}+\frac{e^{-\mathrm{bD}}}{1-e^{-\mathrm{bD}}O_{B}}O_{A}O_{p}}\right)}{b} \end{array}$$

To simplify the calculation, we define:

(A36)
$$O_{k}=\frac{\frac{O_{d}+\frac{1}{1-O_{B}}O_{A}O_{p}}{e^{{\mathrm{ADC}}_{\text{nominal}}b}}-O_{d}}{O_{A}O_{p}}$$

Thus:

(A37)
$$\frac{e^{-\mathrm{bD}}}{1-e^{-\mathrm{bD}}O_{B}}=O_{k}$$

Finally, extract the corrected ADC from this equation:

(A38)
$$D={\mathrm{ADC}}_{\text{correct}}=\frac{\ln\left(1+O_{k}O_{B}\right)-\ln\left(O_{k}\right)}{b}$$
