# Supplementary material for: Simultaneous Assessment of Intracranial Artery and Paravascular CSF Pulsation Using 3D Whole‐Brain Diffusion‐Prepared Cine bSSFP (DECAF) MRI
Source: Magn Reson Med. 2026 Apr 10;96(2):727–40. doi: 10.1002/mrm.70381 (PMC13236401; doi:10.1002/mrm.70381)
Supplement: Supplementary file 1 — Figure S1: Evolution of magnetization during the first 2 diffusion preparation periods in the proposed DECAF sequence. Figure S2: The distribution of the vials in the phantom, along with their measured T1, T2, EPI‐DWI ADC values, and NiCl2 concentrations, T1, T2 values provided in the user manual. Table S1: Demographic information of the participants. Table S2: Arterial wall pulsatility index (PI) and paravascular CSF PI for each artery segment across all subjects. Table S3: ADC maximum and minimum values (×10−3 mm2/s) across the cardiac cycle for each artery segment and each subject. Figure S3: The human brain MRI results of image reconstruction and ADC quantification in one representative subject (movie version of Figure 5). Shown are the diffusion‐prepared cine bSSFP (DECAF) images (A) and quantified ADC map in the whole‐brain CSF regions overlayed on the original images (B). Figure S4: 3D visualization of the paravascular CSF pulsatility index map from one representative participant. (A) Simultaneous visualization of arterial wall and paravascular CSF pulsatility indexes in the 3D space. (B) The layer‐specific paravascular CSF pulsatility index in the 3D space. Figure S5: Relationship of artery wall pulsatility index with age and blood pressure. Figure S6: Relationship of paravascular CSF pulsation with age and Artery Wall Pulsatility Index. Figure S7: A simulation experiment to confirm the AI model can detect cerebral arterial lumen changes. A high‐resolution 2D MPR image (0.25 mm isotropic) of a 2 mm‐diameter artery was used to synthesize cine MRI images. The synthesized images have the same spatial and temporal resolution in this study (0.83 mm isotropic, 16 phases). To simulate the cardiac‐driven lumen changes, the static image was zoomed by a predefined factor to generate an image corresponding to each phase. The process was repeated with multiple ground‐truth pulsatility indices ranging from 0.05 to 0.3. The measured pulsatility indices were calculated by [file MRM-96-727-s001.docx]

**Supporting Information**

Additional Supporting Information may be found in the online version of this article.


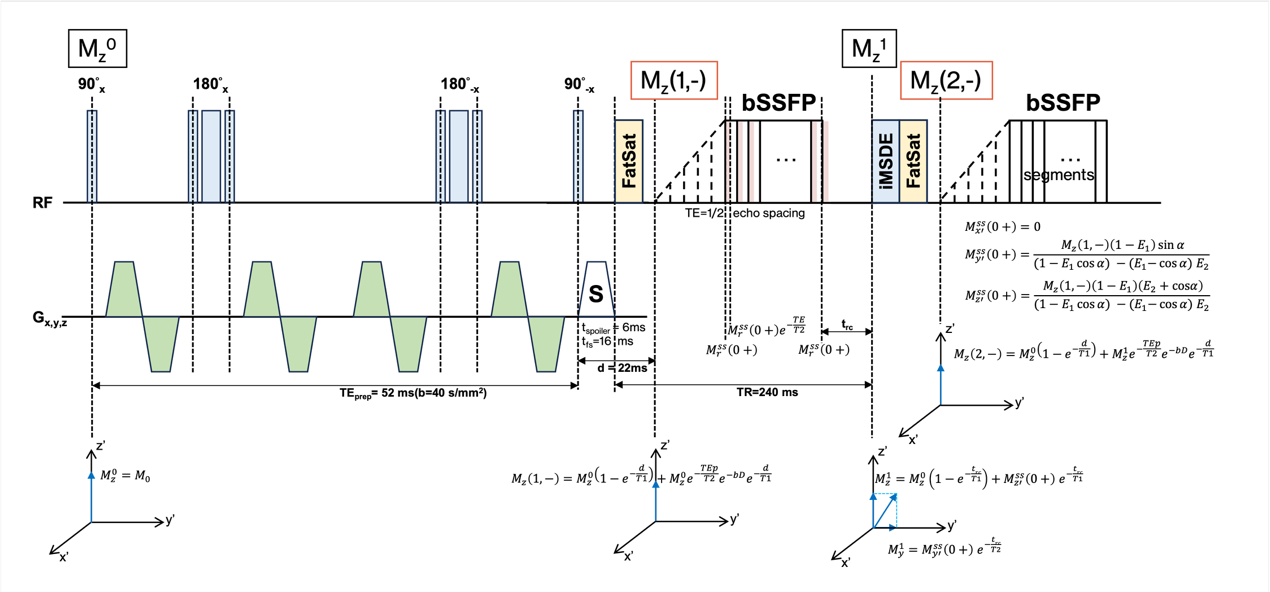


Figure S1: Evolution of magnetization during the first 2 diffusion preparation periods in the proposed DECAF sequence.


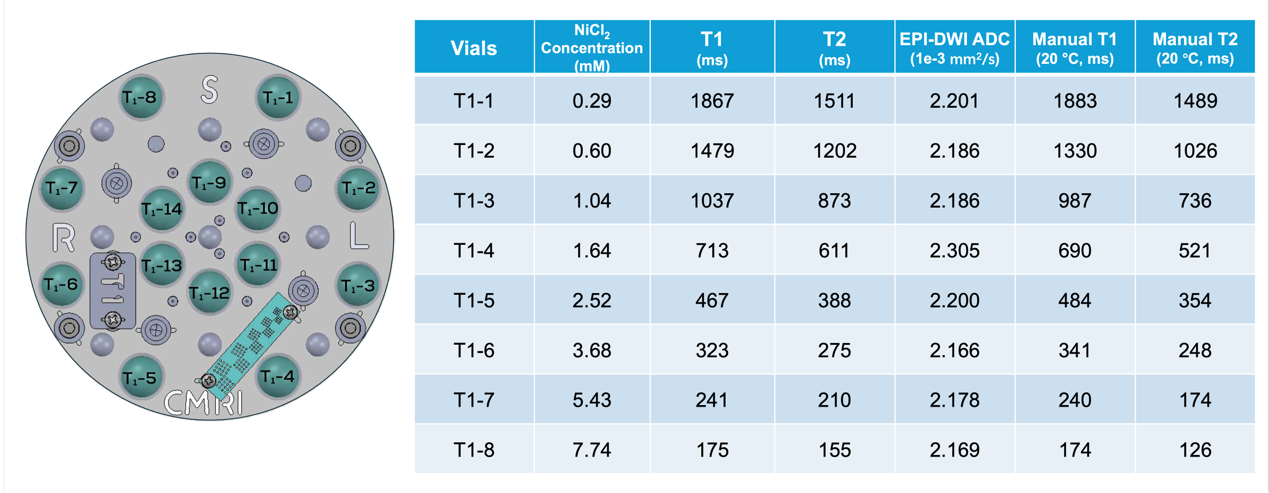


Figure S2: The distribution of the vials in the phantom, along with their measured T1, T2, EPI-DWI ADC values, and NiCl_2_ concentrations, T1, T2 values provided in the user manual.

| **No.** | **Gender** | **Age** |
| --- | --- | --- |
| case1 | Female | 18 |
| case2 | Female | 31 |
| case3 | Female | 40 |
| case4 | Male | 30 |
| case5 | Male | 61 |
| case6 | Male | 71 |

Table S1: Demographic information of the participants.

Table S2: Arterial wall pulsatility index (PI) and paravascular CSF PI for each artery segment across all subjects.

| **Segment** | **A1L** | | **A1R** | | **M1L** | | **M1R** | | **M2L** | | **M2R** | | **P1L** | | **P1R** | |
| --- | --- | --- | --- | --- | --- | --- | --- | --- | --- | --- | --- | --- | --- | --- | --- | --- |
|  | Vessel | CSF | Vessel | CSF | Vessel | CSF | Vessel | CSF | Vessel | CSF | Vessel | CSF | Vessel | CSF | Vessel | CSF |
| case1 | 0.4163 | 0.5148 | 0.3771 | 0.5808 | 0.3199 | 0.4568 | 0.3921 | 0.4885 | 0.3554 | 0.4181 | 0.2452 | 0.4021 | 0.1359 | 0.3990 | 0.2198 | 0.3633 |
| case2 | 0.2040 | 0.4464 | 0.4519 | 0.3839 | 0.2371 | 0.3391 | 0.4485 | 0.3396 | 0.2084 | 0.3379 | 0.2022 | 0.3586 | 0.1805 | 0.3540 | 0.3526 | 0.2763 |
| case3 | 0.3242 | 0.4307 | 0.2603 | 0.4320 | 0.4303 | 0.4108 | 0.1090 | 0.4623 | 0.2916 | 0.3464 | 0.2281 | 0.3461 | 0.1783 | 0.3862 | 0.2556 | 0.4371 |
| case4 | 0.1455 | 0.4404 | 0.1580 | 0.4062 | 0.0997 | 0.3995 | 0.1691 | 0.3911 | 0.1325 | 0.3403 | 0.1547 | 0.3208 | 0.3106 | 0.4641 | 0.2007 | 0.4432 |
| case5 | 0.1066 | 0.3451 | 0.1180 | 0.3068 | 0.1458 | 0.3656 | 0.2570 | 0.2992 | 0.2329 | 0.4394 | 0.2317 | 0.3178 | 0.2569 | 0.4178 | 0.2581 | 0.2965 |
| case6 | 0.1332 | 0.3659 | 0.1502 | 0.3098 | 0.1877 | 0.3512 | 0.1558 | 0.4039 | 0.2445 | 0.3054 | 0.2851 | 0.3779 | 0.2395 | 0.4086 | 0.1381 | 0.3753 |

Table S3: ADC maximum and minimum values (×10^-3^mm²/s) across the cardiac cycle for each artery segment and each subject.

| **Segment** | **A1L** | | **A1R** | | **M1L** | | **M1R** | | **M2L** | | **M2R** | | **P1L** | | **P1R** | |
| --- | --- | --- | --- | --- | --- | --- | --- | --- | --- | --- | --- | --- | --- | --- | --- | --- |
|  | Max | Min | Max | Min | Max | Min | Max | Min | Max | Min | Max | Min | Max | Min | Max | Min |
| case1 | 13.32 | 10.63 | 10.92 | 7.95 | 14.31 | 12.27 | 15.88 | 11.64 | 15.38 | 12.13 | 14.81 | 11.99 | 16.45 | 12.78 | 15.55 | 12.93 |
| case2 | 9.26 | 8.27 | 10.89 | 9.73 | 15.64 | 13.16 | 12.66 | 11.04 | 18.09 | 15.22 | 13.83 | 11.49 | 11.55 | 10.33 | 11.88 | 10.76 |
| case3 | 9.95 | 8.54 | 10.07 | 7.85 | 15.12 | 12.50 | 12.08 | 9.10 | 13.20 | 11.50 | 17.01 | 13.03 | 13.15 | 11.48 | 12.06 | 9.87 |
| case4 | 8.37 | 7.27 | 8.14 | 6.98 | 10.50 | 8.62 | 9.78 | 8.60 | 18.18 | 14.04 | 12.41 | 10.49 | 14.04 | 10.73 | 17.72 | 12.71 |
| case5 | 14.63 | 12.61 | 14.25 | 12.52 | 13.98 | 12.27 | 14.54 | 12.20 | 15.74 | 11.76 | 16.24 | 13.31 | 16.28 | 12.35 | 13.30 | 11.78 |
| case6 | 15.78 | 14.04 | 14.28 | 13.13 | 14.96 | 14.04 | 16.59 | 13.45 | 13.48 | 12.50 | 13.75 | 11.21 | 15.23 | 12.37 | 11.55 | 10.23 |

Figure S3: The human brain MRI results of image reconstruction and ADC quantification in one representative subject (movie version of Figure 5). Shown are the diffusion-prepared cine bSSFP (DECAF) images (A) and quantified ADC map in the whole-brain CSF regions overlayed on the original images (B).


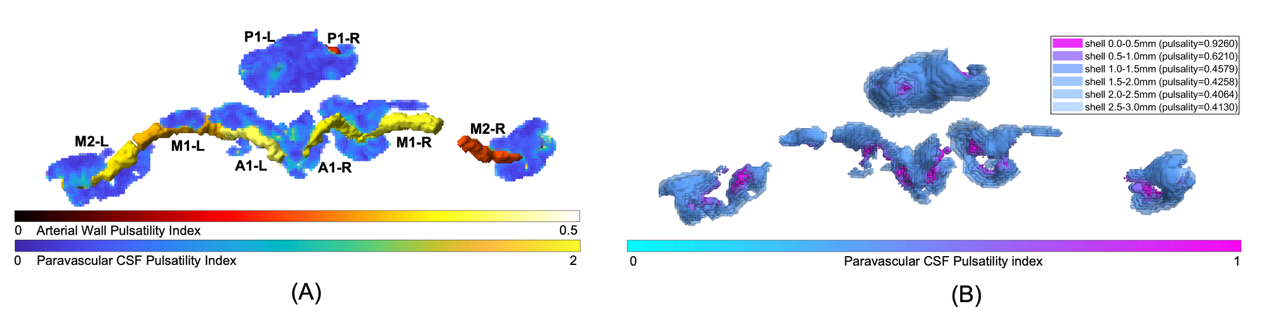


Figure S4: 3D visualization of the paravascular CSF pulsatility index map from one representative participant. (A) Simultaneous visualization of arterial wall and paravascular CSF pulsatility indexes in the 3D space. (B) The layer-specific paravascular CSF pulsatility index in the 3D space.


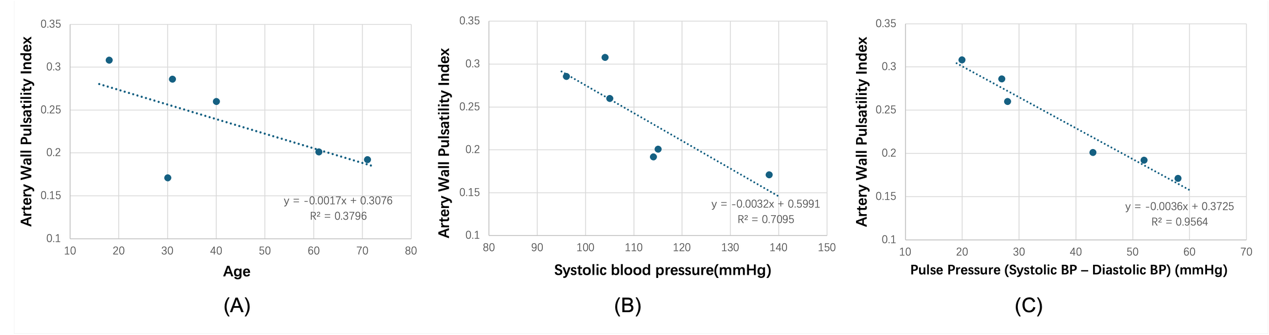


Figure S5: Relationship of artery wall pulsatility index with age and blood pressure.


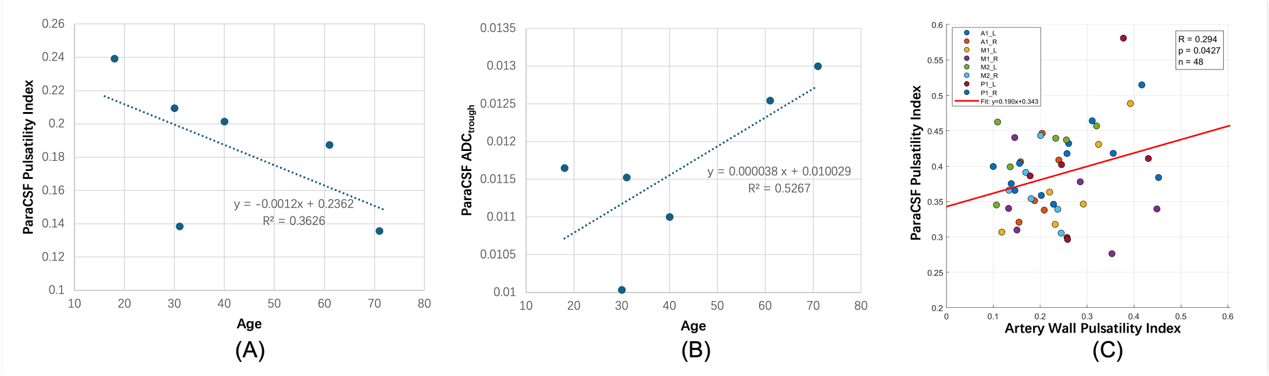


Figure S6: Relationship of paravascular CSF pulsation with age and Artery Wall Pulsatility Index.


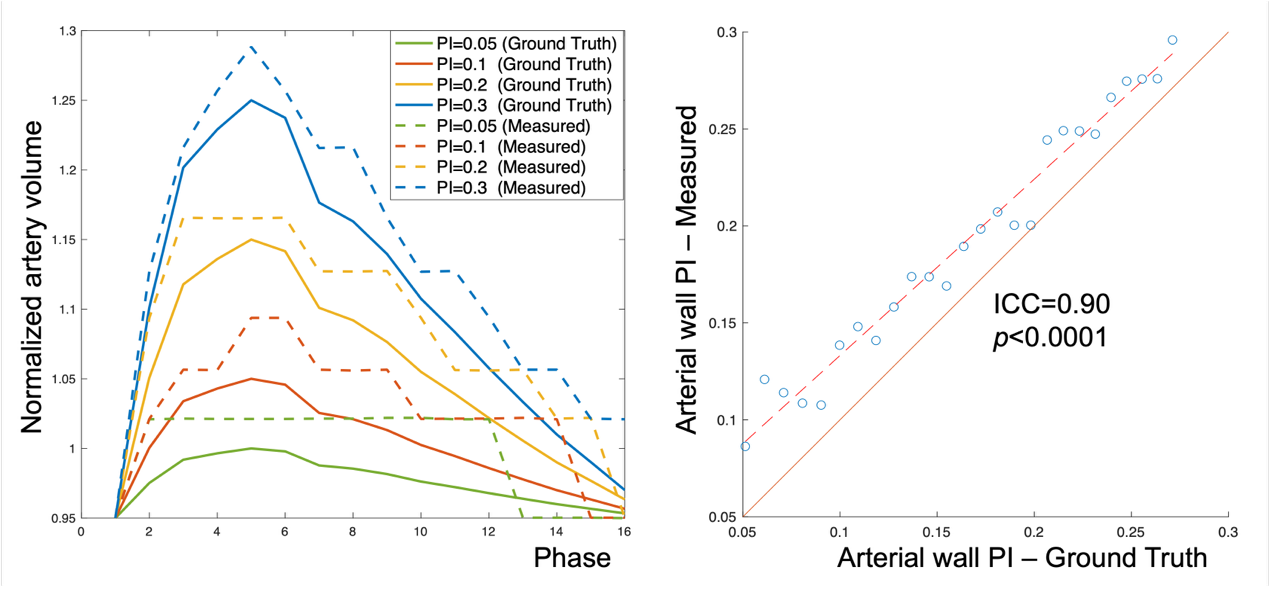


Figure S7: A simulation experiment to confirm the AI model can detect cerebral arterial lumen changes. A high-resolution 2D MPR image (0.25 mm isotropic) of a 2 mm-diameter artery was used to synthesize cine MRI images. The synthesized images have the same spatial and temporal resolution in this study (0.83 mm isotropic, 16 phases). To simulate the cardiac-driven lumen changes, the static image was zoomed by a predefined factor to generate an image corresponding to each phase. The process was repeated with multiple ground-truth pulsatility indices ranging from 0.05 to 0.3. The measured pulsatility indices were calculated by segmenting synthesized cine images using the ONet model. Though the measured volume changes were overestimated (likely because the measured changes were rounded by voxel size), they can well characterize the ground-truth waveforms. The measured and ground-truth pulsatility indices have a good consistency (ICC = 0.90).
